# Advances in the role of extracellular vesicles in circulating microRNA biomarker discovery for lung cancer

**DOI:** 10.3389/fcell.2025.1676530

**Published:** 2025-11-12

**Authors:** Ayaz Belkozhayev, Minnatallah Al-Yozbaki, Yeldar Ashirbekov, Kantemir Satken, Arman Abaildayev, Askar Yeleussizov, Nurlan Jainakbayev, Kamalidin Sharipov, Cornelia M. Wilson

**Affiliations:** 1 Department of Chemical and Biochemical Engineering, Geology and Oil-Gas Business Institute Named After K. Turyssov, Satbayev University, Almaty, Kazakhstan; 2 Structural and Functional Genomics Laboratory of M.A. Aitkhozhin Institute of Molecular Biology and Biochemistry, Almaty, Kazakhstan; 3 Department of Cancer and Genomics, School of Medical Sciences, College of Medicine and Health, University of Birmingham, Birmingham, United Kingdom; 4 Faculty of Biology and Biotechnology, Al-Farabi Kazakh National University, Almaty, Kazakhstan; 5 Kazakh Institute of Oncology and Radiology, Almaty, Kazakhstan; 6 Department of Public Health, Kazakh-Russian Medical University, Almaty, Kazakhstan; 7 Department of Biochemistry, Asfendiyarov Kazakh National Medical University, Almaty, Kazakhstan; 8 Life Sciences Industry Liaison Lab, School of Psychology and Life Sciences, Canterbury Christ Church University, Discovery Park, Sandwich, United Kingdom; 9 Novel Global Community Educational Foundation, Hebersham, NSW, Australia

**Keywords:** extracellular vesicles, MicroRNAs, lung cancer, liquid biopsy, biomarkers

## Abstract

Lung cancer remains a leading cause of cancer-related mortality worldwide, largely due to late-stage diagnosis and the limited efficacy of current therapeutic approaches. Recent advancements highlight the potential of extracellular vesicles (EVs), particularly those carrying microRNA (miRNA) molecules, as promising non-invasive biomarkers for early detection, prognosis, and therapy monitoring. EVs are nanoscale vesicles secreted by tumour cells, capable of transporting various bioactive molecules including miRNAs while preserving their structural stability in circulation. These miRNAs mirror the molecular state of the tumour and often exhibit distinct expression signatures depending on cancer subtype and stage. Studies have shown that specific EV-associated miRNAs are significantly dysregulated in lung cancer patients and correlate with tumour progression, metastatic potential, and overall survival. Moreover, tracking dynamic changes in EV-miRNA profiles during treatment may provide predictive insights into responsiveness to immunotherapy and targeted therapy. This review emphasizes the diagnostic and prognostic utility of EV-derived miRNAs, highlighting their tumour specificity and stability in bodily fluids. In addition, we summarise key challenges such as the lack of standardisation, EV heterogeneity, and technical variability, while also outlining future directions including single-EV detection, multi-omics integration, AI-driven diagnostics, and therapeutic applications. By integrating these biomarkers into clinical workflows via liquid biopsy, it may become possible to detect lung cancer earlier and adapt therapeutic strategies more effectively ultimately improving patient outcomes and offering new directions in precision oncology.

## 1 Introduction

Lung cancer is one of the most prevalent malignancies worldwide and remains the leading cause of cancer-related mortality (Tang et al., 2025; Bray et al., 2022). According to the latest data from the International Agency for Research on Cancer (IARC) and its Global Cancer Observatory (GLOBOCAN), lung cancer ranks among the top diagnosed cancers and continues to be the leading cause of cancer-related mortality worldwide. In 2022, nearly 2.48 million new lung cancer cases occurred globally, accompanied by approximately 1.8 million deaths (Zhou et al., 2024). The age-standardised incidence rate (ASIR) was estimated at 23.6 per 100,000 people, and the age-standardised mortality rate (ASMR) was around 16.8 per 100,000 people (Guo et al., 2024). Projections suggest that if current trends persist, by 2050 the burden of lung cancer could rise substantially, potentially reaching ∼4.62 million new cases and ∼3.55 million deaths annually (Zhou et al., 2024).

Due to the typically late manifestation of clear clinical symptoms, lung cancer is frequently diagnosed at advanced stages, thereby reducing the effectiveness of therapeutic interventions and markedly deteriorating patient survival outcomes (Polanco et al., 2021; Vicidomini, 2023). Among the emerging avenues in cancer research is the investigation of tumour-derived circulating microRNAs (miRNAs) and extracellular vesicles (EVs) as non-invasive biomarkers within the concept of liquid biopsy (Zhou et al., 2023). These entities encapsulate tumour-specific molecular signatures, thereby enabling early disease detection and dynamic assessment of treatment response (Ma et al., 2025). EVs are nanoscale, membrane-bound vesicle-like particles released by cells (Belkozhayev et al., 2022; Wen et al., 2024). They carry a diverse cargo of biomolecules, including proteins, lipids, DNA, and various types of RNA particularly miRNAs (Dellar et al., 2022; Babuta and Szabo, 2022; Chen T. et al., 2025). EVs play a critical role in intercellular communication and act as carriers of molecular signals involved in the pathogenesis of various diseases, including cancer (Godakumara et al., 2022). The molecular cargo delivered via EVs serves as a crucial mediator of intercellular signalling, contributing to the regulation of various physiological and pathological processes, including tumorigenesis (Kumar et al., 2024; Kuang et al., 2025). In the context of cancer, tumour-derived EVs can transmit oncogenic signals to surrounding cells within the tumour microenvironment, enhancing their capacity for proliferation, migration, and angiogenesis (Najaflou et al., 2022). Moreover, several miRNAs transported by EVs have been experimentally shown to promote the invasive and metastatic potential of cancer cells (Martellucci et al., 2020). A key biological feature of EVs is their ability to protect nucleic acids particularly miRNAs from enzymatic degradation in the extracellular environment (Buzas, 2023). This protective capacity allows EV-associated miRNAs to remain stable for extended periods in biological fluids such as blood, thereby enabling their direct analysis through non-invasive approaches (Ma et al., 2023; Ferro et al., 2024). The presence of disease-specific expression patterns and the inherent stability of EV-carried miRNAs pave the way for their use as reliable diagnostic and prognostic biomarkers in lung cancer (Bartoszewska et al., 2025). In recent years, there has been a marked increase in interest toward the investigation of circulating EVs as reliable biomarkers for lung cancer (Vasu et al., 2025; Ge et al., 2023). Numerous studies have demonstrated that the EV-associated miRNA expression profiles in the plasma of lung cancer patients differ significantly from those of healthy donors (Li et al., 2024a; Gao et al., 2022). Moreover, the expression levels of certain EV-derived miRNAs have been found to correlate strongly with clinical tumour stage, treatment response, and overall patient survival (Mok et al., 2024; Seyhan, 2023). For instance, diagnostic models based on plasma EV-miRNA signatures have been proposed to distinguish between malignant and benign pulmonary nodules (Liu et al., 2023). One pilot study even indicated that EV-miRNA expression patterns could predict the malignancy of nodules smaller than 1 cm in diameter (Paterson et al., 2022). Collectively, these findings highlight the high diagnostic potential of EV-encapsulated miRNAs for the early detection of lung cancer and for improving clinical prognostication (Paterson et al., 2022; Eckhardt et al., 2023). Previous reviews have mainly focused on the diagnostic potential of EV-miRNAs in liquid biopsy. This review advances the field by integrating updated evidence on EV–miRNA–mediated signaling and tumour progression, while also highlighting unresolved challenges such as heterogeneity and lack of standardisation. Moreover, this review evaluates emerging profiling technologies including single-EV detection, multi-omics approaches, and AI-driven analytics. In addition, it explores the role of EVs and miRNAs in the early diagnosis and prognostication of lung cancer, providing an overview of EV types and mechanisms, a critical evaluation of isolation techniques and miRNA profiling methods, and a synthesis of their biomarker potential with prospective clinical and therapeutic applications.

## 2 EVs: types, composition, and mechanisms of action

### 2.1 Types of EVs

EVs are microscopic, vesicle-like structures released by cells into the extracellular environment. They carry a diverse range of biomolecules, including proteins, lipids, and nucleic acids (Di Bella, 2022). EVs serve as essential mediators of intercellular communication within the body, enabling the transfer of biological information from one cell to another (Petroni et al., 2023; Wessler and Meisner-Kober, 2025). Currently, the roles of EVs in both physiological and pathological processes particularly in tumour development and the modulation of immune responses are being extensively investigated (Reale et al., 2022; Gong et al., 2025).

The main types of EVs include exosomes, microvesicles, and apoptotic bodies (Lee et al., 2022). Exosomes represent the smallest EV subpopulation, with a diameter of approximately 30–150 nm, also known as small EVs (SEV) (Zarovni et al., 2025). They are formed through the endosomal pathway as intraluminal vesicles within multivesicular bodies (MVBs). When MVBs fuse with the plasma membrane, these vesicles are released into the extracellular space as exosomes (Xu D. et al., 2022; Kalluri and LeBleu, 2020; Visnovitz et al., 2025). Microvesicles (also known as medium EVs) are EVs ranging from approximately 100–1,000 nm in diameter and are generated by direct outward budding from the plasma membrane (Singh B. et al., 2025). In contrast, apoptotic bodies (also known as large EVs (LEV)) represent the largest type of EVs, released from cells undergoing apoptosis. Their size typically ranges from 1 to 5 μm, and they may contain cellular fragments and nuclear material (Huang et al., 2024; Gregory and Rimmer, 2023).

However, the distinction between these EV subtypes remains challenging, as their size ranges often overlap and many markers are not exclusive to a single population (Verweij et al., 2021; Jeppesen et al., 2019). This lack of clear separation complicates both experimental characterization and clinical translation. In lung cancer research, refining EV classification is critical because different vesicle subtypes show distinct capacities for carrying oncogenic or tumour-suppressive miRNAs, which directly influences their diagnostic and prognostic potential. Recent advances such as single-vesicle imaging, high-resolution flow cytometry, and nanoplasmonic detection are being developed to better distinguish EV subtypes and capture their biological heterogeneity (Kobayashi et al., 2024; von Lersner et al., 2024).

### 2.2 Composition of EVs

**FIGURE 1:**
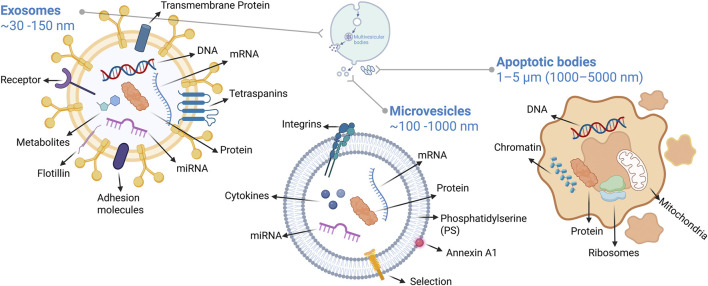
Structural and molecular composition of EVs including exosomes microvesicles and apoptotic bodies. Created with BioRender, License No. SQ28D9D1PG.

EVs contain a diverse array of biomolecules, including proteins, lipids, nucleic acids (DNA and RNA), and metabolites (Jin et al., 2025). Their membrane consists of a double phospholipid bilayer that protects the internal cargo from the extracellular environment. This membrane features specific proteins derived from the donor cell’s plasma membrane and endosomal system, such as tetraspanins, integrins, and receptors (Hallal et al., 2022; Jankovičová et al., 2020). The EV lumen is enriched with cytosolic proteins, various RNA species, and, in some cases, DNA fragments. This molecular heterogeneity reflects the complex and multifaceted roles of EVs in intercellular communication (Malkin and Bratman, 2020; Huang et al., 2025). Figure 1 illustrates the structural and molecular composition of extracellular vesicles, including exosomes, microvesicles, and apoptotic bodies, and highlights their distinct sizes and cargo profiles.

The protein composition of EVs is heterogeneous and includes tetraspanins (CD9, CD63, CD81, CD82), adhesion molecules (integrins, EpCAM, MHC-I/II, ICAM-1), ESCRT proteins (TSG101, ALIX, Rab GTPases), and heat shock proteins (Hsp70, Hsp90) (Stajano et al., 2023; Jankovičová et al., 2024). Although exosomes and microvesicles show differences in proteomic profiles (e.g. CD63/CD81 vs. Annexin A1/CD9), these markers are not absolute (Jeppesen et al., 2023; De Sousa et al., 2023).

Nucleic acids are a key component of EV cargo. EVs contain diverse RNA species, including mRNAs, miRNAs, lncRNAs, and circRNAs (Kwok et al., 2021; Miceli et al., 2024). Notably, miRNAs can constitute up to 40% of total RNA reads in plasma-derived EVs (Mittelbrunn et al., 2011). For instance, tumour-derived EVs carrying miR-9 activate the JAK/STAT signalling cascade in endothelial cells by suppressing SOCS5, thereby promoting angiogenesis. Similarly, EV-miR-23a, part of the miR-23a/27a/24-2 cluster, has been shown to drive postoperative progression and metastasis in early-stage NSCLC by activating Wnt/β-catenin signalling and inducing epigenetic silencing of p16 and CDH13 (Fan et al., 2021). In addition, EV-miR-105 contributes to invasion and vascular remodelling within the tumour microenvironment (Chatterjee et al., 2020). Beyond RNA species, EVs can also carry DNA fragments including genomic, mitochondrial, and extrachromosomal circular DNA (eccDNA) that mirror donor cell mutations and are emerging as promising biomarkers for cancer detection and monitoring (Qian H. et al., 2024).

The lipid composition of EVs defines their structure and function, being rich in cholesterol, sphingomyelin, glycosphingolipids, and ceramide (Zhou et al., 2017). These lipids regulate vesicle budding and stability, and tumour EVs enriched in sphingolipids can modulate immune responses (Pathania et al., 2021). Overall, the molecular composition of EVs mirrors their cellular origin, with miRNA cargo in particular emerging as a critical signature for lung cancer diagnostics and therapeutic targeting (Salomon et al., 2022; Amin et al., 2024).

### 2.3 Mechanisms of extracellular vesicle release

**FIGURE 2:**
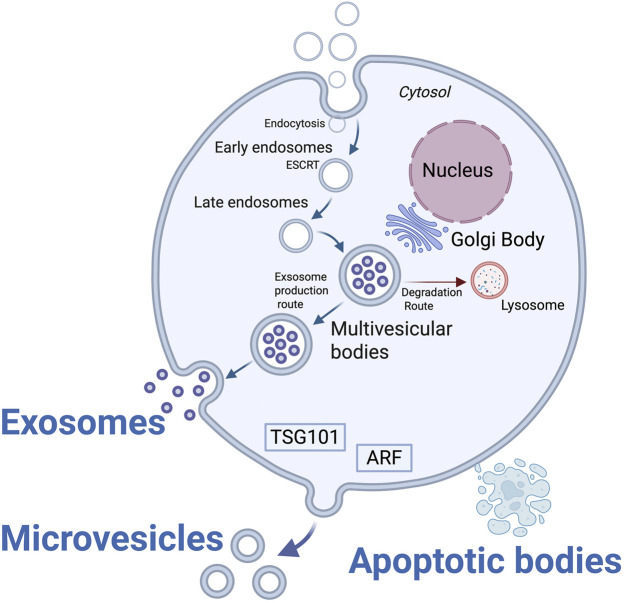
Biogenesis Pathways of Exosomes and Microvesicles. Created with BioRender, License No. UX28DAB76O. Distinct intracellular mechanisms underlie the formation of exosomes and microvesicles. Exosomes are generated as intraluminal vesicles within MVBs and are released following MVB fusion with the plasma membrane via ESCRT-dependent or ESCRT-independent pathways. Microvesicles originate by direct outward budding of the plasma membrane, regulated by cytoskeletal remodeling, calcium signalling, phospholipid asymmetry loss, and involvement of ARF6, TSG101, and related molecules. Apoptotic bodies arise during programmed cell death (apoptosis) through membrane blebbing and cell fragmentation, and typically contain nuclear fragments, cytoplasmic components, and organ.

The biogenesis and secretion of EVs are subtype-specific and occur through tightly regulated intracellular pathways. Exosomes originate from the endosomal system via inward budding of the endosomal membrane, resulting in the formation of intraluminal vesicles (ILVs) within MVBs (Dixson et al., 2023; Kang et al., 2021; Teng and Fussenegger, 2020; Battistelli and Falcieri, 2020; Gurung et al., 2021). When MVBs fuse with the plasma membrane, ILVs are secreted into the extracellular space as exosomes (Krylova and Feng, 2023; Palomar-Alonso et al., 2023) (Figure 2). This process is primarily governed by the endosomal sorting complex required for transport (ESCRT) machinery, although ESCRT-independent mechanisms involving ceramide synthesis and tetraspanin clustering have also been identified (Jin et al., 2022; Liu et al., 2024). Exosome release is facilitated by Rab GTPases and SNARE complexes, which regulate MVB trafficking and membrane fusion events (Ovčar and Kovačič, 2025). Pathological conditions such as hypoxia and oncogenic signalling increase exosome release, enriching them with tumour-derived miRNAs (Kenific et al., 2021; Zubkova et al., 2024). Microvesicles are EVs formed by the direct outward budding of the plasma membrane (Chatterjee et al., 2024).

Apoptotic bodies (ApoBDs) are LEVs formed during programmed cell death because of cell fragmentation (Kakarla et al., 2020). These vesicles may contain remnants of the nucleus, mitochondria, and other organelles, as well as chromatin DNA. ApoBDs are typically enriched in nuclear DNA and chromatin fragments bound to histones, and they display strong external expression of phosphatidylserine (Qian Y. et al., 2024; Singh M. et al., 2025).

The biogenesis and secretion of EVs are tightly regulated processes that adapt to the physiological state of the cell and external stimuli (Erwin et al., 2023). Factors such as cellular stress, hypoxia, elevated intracellular calcium levels, and oncogenic activity are known to enhance exosome and microvesicle release (Gurunathan et al., 2021). Recent advances have highlighted that EV biogenesis is not solely governed by classical ESCRT pathways but also intersects with major intracellular signaling cascades such as mTOR, p53, NF-κB, and MAPK, which dynamically respond to stress and oncogenic activation (Phan et al., 2024; Yeat and Chen, 2025; Huang et al., 2023).

### 2.4 Role of EVs in tumour progression and intercellular communication

EVs, including exosomes and microvesicles, are actively utilised as key mediators of communication between cancer cells and their surrounding microenvironment (Kalluri and McAndrews, 2023). These nanoscale structures transport proteins, nucleic acids (miRNA, mRNA, lncRNA), lipids, and metabolites, delivering tumour-specific molecular information to recipient cells (Liu et al., 2022; Wang Y. et al., 2023; Samidurai et al., 2023).

Recent studies have further clarified the multifaceted role of EVs in cancer pathogenesis. For instance, Hánělová et al. (2024) demonstrated that EVs released by tumour cells facilitate molecular communication between cancer cells and their surrounding microenvironment (Hánělová et al., 2024; Hanelova et al., 2023). This, in turn, promotes fibroblast activation, tissue remodelling, immune suppression, angiogenesis, formation of the pre-metastatic niche, and enhanced metastatic progression indicating that EVs actively support key components of tumour-associated homeostasis (Caligiuri and Tuveson, 2023; Patras et al., 2023).

EVs can enhance tumour cell proliferation by transporting cancer-specific signalling molecules (Zhang et al., 2021). For example, a study by Patel et al. (2024) demonstrated that EVs derived from non-small cell lung cancer (NSCLC) cells deliver α-SMA (alpha-smooth muscle actin) to surrounding lung fibroblasts and NSCLC cells, thereby promoting cell proliferation and reducing apoptosis levels (Patel et al., 2024).

Additionally, EVs derived from a human colorectal cancer cell model have been shown to enhance tumour cell proliferation and viability by activating the MAPK/ERK signalling pathway (Long et al., 2024; Rahmati et al., 2024). These findings support the notion that EVs promote tumour growth by delivering mitogenic and survival factors (Lopez et al., 2023).

EVs support tumour progression by promoting cancer-associated angiogenesis, the formation of new blood vessels (Ateeq et al., 2024; Vader et al., 2014). For instance, EVs derived from glioblastoma cells have been found to carry highly angiogenic factors such as VEGF, TGF-β, IL-6, and IL-8 (Yekula et al., 2020; Russo et al., 2023). These proteins activate endothelial cells and enhance the delivery of oxygen and nutrients to the tumour. Moreover, EVs from lung adenocarcinoma cells have been shown to stimulate neovascularisation through the delivery of the sortilin protein, which facilitates the transfer of angiogenic molecules like VEGF and IL-8 to endothelial cells (Yang et al., 2025; Palazzo et al., 2022).

EVs play a pivotal role in enhancing the metastatic potential of cancer cells (Chang et al., 2021). By transporting specific molecules that promote the formation of pre-metastatic niches in distant tissues, EVs facilitate tumour dissemination (Giusti et al., 2023). As demonstrated by Li et al. (2024b), tumour-derived EVs can direct organotropic signalling, influencing metastatic organotropism and even modulating the microbial composition of distant tissues (Li et al., 2024b). These vesicles deliver molecules such as, proliferative cytokines, and immunosuppressive factors, which collectively promote, stromal remodelling, and immune suppression in target organs thus creating a favourable microenvironment for metastasis. In this way, EVs act as active mediators that lay the groundwork for metastatic progression (Chen T. et al., 2025; Wiklander et al., 2019).

EVs play a crucial role in enabling cancer cells to evade immune surveillance (Yue et al., 2023). Specifically, tumour-derived EVs often exhibit elevated expression of the immune checkpoint molecule PD-L1, which binds to the PD-1 receptor on T lymphocytes and suppresses immune responses. As a result, EVs facilitate immune evasion by tumour cells and may contribute to reduced efficacy of anti-tumour immunotherapies (Ye et al., 2024; Ahmadi et al., 2024). Moreover, recent evidence by Padinharayil et al. (2025) demonstrated that lung cancer–derived small extracellular vesicles (sEVs) profoundly alter T-cell cytokine expression and protein profiles, leading to reduced T-cell viability and dysregulated apoptosis and inflammatory pathways. While some cytotoxic markers remain upregulated, the overall signalling balance suggests impaired T-cell activation. These findings underscore that tumour-derived EVs do not merely suppress immune surveillance through PD-L1 expression but also reprogram T-cell functional states at the transcriptomic and proteomic level, highlighting their dual role in immune evasion and tumour–immune crosstalk (Padinharayil et al., 2025). Beyond their roles in proliferation, angiogenesis, and immune evasion, recent studies reveal that tumour-derived EVs can also drive epigenetic reprogramming through the transfer of non-coding RNAs (Zhang et al., 2025), reshape cellular metabolism by modulating glycolysis and lipid pathways, and even influence the tumour-associated microbiome to favour metastasis. Moreover, EVs have been implicated in resistance to next-generation immunotherapies by carrying additional immune checkpoint ligands such as TIM-3 and LAG-3 (Kumar et al., 2024). Importantly, emerging clinical trials are now testing EV-derived RNA and protein signatures as predictive biomarkers of therapy response in NSCLC patients (Guo et al., 2023), underscoring their translational potential.

Numerous studies have shown that EVs contribute to the development of resistance to chemotherapy and targeted therapies (Zheng et al., 2024; Fu et al., 2023). Notably, tumour-derived EVs may carry drug resistance related proteins such as P-glycoprotein, which, when delivered to recipient cells, enhance the efflux of cytotoxic agents. For example, in melanoma cells with elevated salicylate levels, P-glycoprotein transferred via EVs has been shown to increase chemoresistance (Fu et al., 2023; Kingreen et al., 2024). These findings highlight the role of EVs as “protective carriers” that mediate drug resistance mechanisms and potentially reduce treatment efficacy (Chen Y. et al., 2025). EVs facilitate tumour metastasis by “priming” target tissues prior to metastatic colonisation (Ghoroghi et al., 2021). They influence stromal cells in distant organs, promoting the formation of a low-immunity, pro-tumorigenic microenvironment. For instance, tumour-derived EVs have been shown to activate macrophages in the bone marrow and lungs, thereby creating favourable conditions for the settlement of metastatic cancer cells (Wang Y. et al., 2023; Wang et al., 2024). In this way, EVs play a decisive role in the establishment of pre-metastatic niches, structurally and functionally supporting the metastatic cascade (Izhar and Lesniak, 2025). Table 1 summarises the molecules transported by EVs associated with different cancer types, their effects on target cells, and the resulting biological outcomes. For instance, EVs derived from lung cancer carry the PD-L1 molecule, which suppresses T-lymphocyte activity, while EVs from breast cancer deliver Annexin II, promoting angiogenesis in endothelial cells (Sato and Weaver, 2018; Abdul-Rahman et al., 2024). In summary, tumour-derived EVs function as multifaceted regulators that not only promote proliferation, angiogenesis, metastasis, and immune evasion, but also drive metabolic and epigenetic reprogramming, ultimately positioning them as both key facilitators of cancer progression and promising targets for therapeutic intervention.

**TABLE 1:** Characteristics of EVs in different cancer types with their molecular cargo, target cells and biological outcomes.

Cancer type	EV type	Molecular cargo	Target cells	Biological outcome	Reference
Lung cancer (NSCLC)	Exosomes	TGF-β, IL-10, EGFR, miR-210-3p	Epithelial cells, stroma, endothelium	Increased proliferation and migration, EMT, metastasis	Yin et al. (2021), Jiang et al. (2021a), Yang et al. (2020)
Breast cancer	Exosomes	VEGF, miR-105, MMPs, Annexin II	Endothelial cells, fibroblasts	Enhanced angiogenesis, increased vascular permeability, and metastasis	Zhou et al. (2014), Castillo-Sanchez et al. (2022), Khanicheragh et al. (2024)
Colorectal cancer	Exosomes	miR-320d, VEGF	Endothelium, macrophages	Angiogenesis, liver metastasis	Wu et al. (2024)
Pancreatic cancer	Exosomes	miR-5703, miR-616-3p	Endothelium, cancer cells	Activation of PI3K/Akt, enhanced proliferation and angiogenesis	Cao et al. (2021), Chen et al. (2024)
Prostate cancer	Exosomes	Galectin-3, WNT5, Annexin II	Endothelium, stroma	Tumour growth and angiogenesis	Caputo et al. (2020), Stefańska et al. (2023)
Melanoma	Exosomes, microvesicles	PD-L1, Hsp90/IKK complex	Immune cells, endothelium	Immune suppression and angiogenesis	Tang et al. (2022), Li and Liu (2023)

## 3 Circulating EVs and their role in lung cancer diagnosis

Over the past decade, the high mortality rate associated with lung cancer has driven numerous multicentre studies aimed at enhancing early tumour detection through integrated imaging techniques (X-ray, PET, PET/CT) and blood test correlations (Deng et al., 2025). One such study, the 2004 COSMOS trial (COSMOS, 2025), enrolled over 5,000 asymptomatic smokers, individuals at increased risk for lung cancer. Participants were monitored over 5 years with annual low-dose spiral CT scans, blood tests, and spirometry, alongside assessments of the link between COPD and lung cancer. In addition, large-scale studies have explored circulating biomarkers and radiomic data in healthy individuals. For instance, the CLEARLY study (National Cancer Institute, 2025) launched in 2018, is a multifactorial “bio-radiomic” protocol combining imaging data with blood-based biomarkers to improve early lung cancer detection. Radiomic profiles associated with early-stage disease have been linked to molecular and cellular markers such as miRNAs (miRNAs), proteins, circulating tumour cells (CTCs), and EVs. EVs, which participate in cell proliferation, differentiation, and inflammation, have attracted increasing attention not only as biomarkers but also for their therapeutic potential through intercellular communication (Velpula and Buddolla, 2025).

A single miRNA strand can control numerous genes by inhibiting their translation, making them powerful tools for both diagnostics and therapeutics (Condrat et al., 2020). Recent advances include the engineering of EVs with specific ncRNAs, although identifying distinct miRNA signatures in early-stage tumours remains a challenge. Notably, reliance on single time-point measurements risks oversimplifying the evolving clonal architecture of lung cancer. Longitudinal profiling of EV cargo, particularly miRNAs, could address this limitation by capturing dynamic changes in tumour biology and thereby improving early detection and risk stratification. In support of this, Shimada et al. (2023) demonstrated that reduced levels of EV-miR-30d-5p were significantly associated with lymphovascular invasion in early-stage lung adenocarcinoma, underscoring its potential as a prognostic biomarker for identifying patients at higher risk of recurrence. This finding highlights how EV-miRNA signatures can extend beyond diagnostic applications into prognostic stratification, especially in the context of early-stage disease where treatment decisions are most critical.

Taken together, these developments position EVs as more than passive biomarkers. They represent multifunctional theranostic platforms with the potential to unify diagnostic precision, real-time monitoring, and therapeutic delivery. In this way, EVs could redefine the role of liquid biopsy in lung cancer by bridging molecular and imaging domains, ultimately enabling earlier, more personalised, and potentially more effective interventions. Here, we will review highlights in the diagnostic and therapeutic potential of EVs in lung cancer, particularly their application as components of liquid biopsies and theranostic agents.

### 3.1 EVs in the circulation

Understanding the origin of EVs offers key insights into the diverse tissue and cellular sources contributing to the circulating EV population. Notably, an analysis of 101 human plasma samples revealed that 99.8% of circulating EVs are derived from hematopoietic cells, with only 0.2% originating from non-hematopoietic tissues (Li Y. et al., 2020). To trace the sources of EVs, an EV-origin method based on extracellular RNA (exLR) profiles was developed (Li Y. et al., 2020; Shaba et al., 2022). This approach involved several steps, including processing RNA-seq data from various tissues and cell types, constructing and refining signature matrices, selecting and validating predictive models, and mapping the EV origin atlas across normal and disease states using a dedicated algorithm.

In the circulatory system, the majority of EVs originate from platelets. As central players in the haemostatic process, platelets are equipped with various granules that, upon activation by specific stimuli often mediated by components of the complement system release their contents and generate microvesicles (MVs) through outward budding of the plasma membrane (Heijnen et al., 1999; Macey et al., 2011). This mechanism not only underscores the critical function of platelets in coagulation but also highlights their emerging role in intercellular communication within the vasculature.

Beyond platelets, several malignancies, including glioblastoma (GBM), gastric (GC), lung (LC), and skin cancers (SC), have been identified as prolific sources of EVs (Chang et al., 2021; Saber et al., 2020). Platelet-derived EVs, due to their rich and diverse molecular cargo, have been shown to interact dynamically with elements of the tumour microenvironment (TME), contributing to cancer progression, remodelling of the TME, and facilitation of metastatic spread (Cho, 2021). The circulating EV pool is further enriched by contributions from various immune and vascular cell types, including monocytes, macrophages, dendritic cells, natural killer (NK) cells, B and T lymphocytes, megakaryocytes, and endothelial cells (Raposo et al., 1996; Ren et al., 2011; Żmigrodzka et al., 2016). In contrast, tissues such as adipose, skeletal muscle, and cardiac tissue release comparatively lower quantities of EVs under physiological conditions (Chaturvedi et al., 2015; Ogawa et al., 2010).

Importantly, cancer cells actively secrete EVs not only into the bloodstream but also into local tissue fluids, enhancing their potential utility as diagnostic biomarkers (Melo et al., 2015). This abundant and specific release of EVs supports their application in non-invasive cancer diagnostics and disease monitoring. Accordingly, ongoing investigations into the tissue-specific origins and molecular heterogeneity of EVs hold great promise for advancing our understanding of cellular diversity and improving the precision of diagnostic strategies (Yáñez-Mó et al., 2015).

EVs have attracted great interest as circulating biomarkers in lung cancer. Lung cancer is often diagnosed late, so there is an intense effort to find non-invasive markers for early detection. EVs are of particular interest because they are more numerous and intrinsically more stable than other liquid biopsy analytes (e.g. cell-free DNA or circulating tumour cells) (Castro-Giner et al., 2018; Caputo et al., 2023). The lipid bilayer protects EV contents (including RNA) from enzymatic degradation in blood. In fact, it has been demonstrated that EV membranes preserve miRNAs under harsh conditions (extreme pH or RNases) far better than free miRNA alone (Russell et al., 2019). For these reasons, EVs are considered ideal vehicles for carrying tumour-derived molecular signals into circulation. Tumour-derived EVs in the blood can thus serve as a “liquid biopsy” of the tumour, carrying on their surface or within their lumen a snapshot of the tumour’s molecular state. Recent reviews note that EV-miRNAs have already been recognised as robust biomarkers in various cancers, assisting in diagnosis and prognosis. In lung cancer specifically, EVs are being actively pursued as diagnostic tools because they can reveal oncogenic mutations and gene expression changes (including miRNAs) non-invasively.

Multiple studies report that cancer patients have elevated levels of circulating EVs relative to healthy individuals, with EV counts often increasing with tumour stage (Caputo et al., 2023). For instance, one study found that EV levels in pulmonary blood correlated strongly with NSCLC clinical stage (Sohal and Kasinski, 2023). Tumour cells can secrete tens of thousands of EVs per day approximately 20,000 vesicles per cell within 48 h a figure corroborated by quantitative studies reporting medulloblastoma cells release between 13,400 and 25,300 EVs per cell during the same period (Choi et al., 2020; Balaj et al., 2011).

Importantly, EVs carry tumour-specific molecules (mutant EGFR, KRAS, PD-L1, oncogenic miRNAs, etc.) that reflect the molecular status of the tumour. Comparative analyses have found distinct profiles of EV cargo in lung cancer patients versus controls, for example, specific EV miRNA signatures differ significantly between NSCLC patients and healthy subjects. Functionally, circulating EVs from lung tumours can reprogram recipient cells: they transfer oncogenic proteins and RNAs that alter the tumour microenvironment (TME) and pre-metastatic niches (Li Y. et al., 2020; Han et al., 2024). Owing to their exceptional stability, tumour-specific miRNA profiles, and ability to transfer oncogenic cargo that reprograms recipient cells, circulating EVs represent heterogeneous yet informative biomarkers that reflect the tumour’s molecular landscape and support both diagnosis and prognosis in lung cancer (Ciferri et al., 2021; Sohal and Kasinski, 2023).

### 3.2 Functional role of EVs in cancer

Beyond their biomarker value, EVs play active roles in lung cancer biology. Tumour-derived EVs promote angiogenesis, invasion and immune evasion. For example, lung cancer exosomes can carry immune-suppressive signals that inhibit CD8^+^ T cells and natural killer cells, and they can reprogram stromal or bone marrow cells to create a permissive metastatic niche (Abdul Manap et al., 2024; Carreca et al., 2024). Moreover, EVs also shuttle oncogenic proteins and RNAs; for instance, EVs from NSCLC patients were found to carry EGFR mutations and oncogenic ALK–EML4 fusions, potentially enabling detection of driver mutations from blood (Rabinowits et al., 2009; Rolfo et al., 2017; Yanaihara et al., 2006). Thus, EVs mediate cell–cell communication in the tumour microenvironment and beyond. They transfer functional cargo (mRNAs, noncoding RNAs and proteins) that can reprogram recipient cells for example, altering gene expression, promoting proliferation or conferring drug resistance (Sun and Chang, 2024; Prieto-Vila et al., 2021).

All cells including lung cancer cells constitutively secrete EVs (Saviana et al., 2021). Cancer cells may release even more EVs than normal cells due to oncogenic stress. EVs from lung tumours enter the bloodstream through leaky tumour vasculature and drainage from lung parenchyma (Bebelman et al., 2021; Xu D. et al., 2022). Notably, EVs have been detected in bronchoalveolar lavage fluid and pleural effusions of lung cancer patients as well as in peripheral blood. Because EVs mirror the molecular composition of the donor cells, blood EVs can carry tumour-specific signatures (proteins, RNAs) even in early-stage disease (Liam et al., 2020; Tang et al., 2023). In summary, EVs are ubiquitous in circulation and serve as carriers of tumour-derived information.

### 3.3 EV-mediated transfer of miRNAs

**FIGURE 3:**
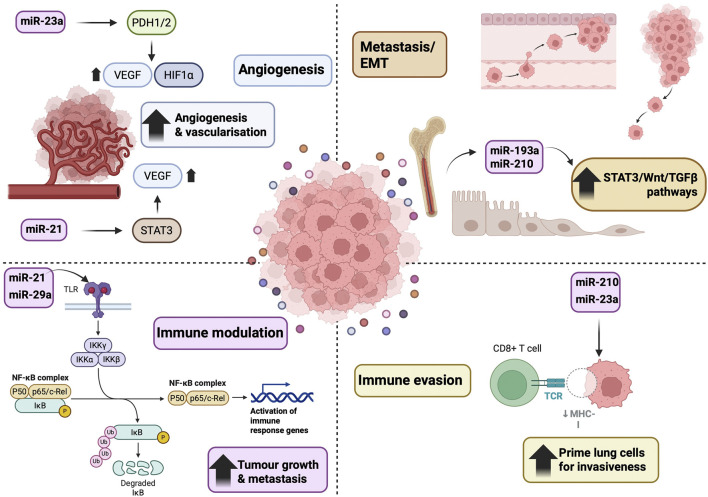
EVs released by tumour cells deliver miRNAs to recipient cells, modulating lung cancer progression through multiple pathways. (1) Immune modulation: Tumour-derived EVs carrying miR-21/miR-29a activate NF-κB via TLR7/8 on immune cells, promoting inflammation and tumour growth. (2) Immune evasion: Hypoxic NSCLC-derived EVs enriched in miR-210/miR-23a suppress NK cell cytotoxicity, aiding immune escape. (3) Angiogenesis: EV miR-23a and miR-21 enhance VEGF expression via HIF-1α stabilisation and STAT3 activation, respectively (4) Metastasis and EMT: EV miRNAs (e.g. miR-193a, miR-210) and transcription factors (e.g. SNAI1, ZEB1) promote epithelial–mesenchymal transition and invasiveness through STAT3, Wnt, and TGF-β signalling.

EVs selectively package and shuttle miRNAs as signalling cargo. Packaging is an active, regulated process: specific RNA-binding proteins recognise sequence motifs on miRNAs to sort them into EVs. For example, the RBP hnRNPA2B1 (when sumoylated) binds “EXO-motifs” in certain miRNAs, loading them into exosomes (Li C. et al., 2021). This selectivity means exosomal miRNA profiles often differ markedly from parent cells. Other mechanisms, for example, ceramide-dependent secretion or Ago2-associated loading have also been implicated, reflecting the complex regulation of EV cargo. Once released, EVs can deliver their miRNA payload to diverse recipient cells, modulating cancer and host pathways (Arora and Verma, 2024; Albanese et al., 2021). In lung cancer, key examples include (Figure 3).

Immune modulation: Tumour-derived EVs containing miR-21 and miR-29a bind Toll-like receptors (TLR7/8) on immune cells, triggering an NF-κB–mediated inflammatory cascade (↑TNFα, IL-6) that promotes tumour growth and metastasis (Fabbri et al., 2012).

Immune evasion: Hypoxic NSCLC cells secrete EVs enriched in miR-210 and miR-23a, which suppress natural killer (NK) cell cytotoxicity (e.g. downregulating CD107a on NK cells) and aid immune escape (Berchem et al., 2016).

Angiogenesis: EV miR-23a (upregulated under hypoxia) targets endothelial prolyl hydroxylases (PHD1/2), stabilising HIF-1α and VEGF to enhance angiogenesis. Similarly, EV miR-21 activates STAT3 signalling in lung epithelial/endothelial cells, upregulating VEGF and promoting blood vessel formation (Hu et al., 2019).

Metastasis/EMT: EV miRNAs can induce epithelial–mesenchymal transition and migration. For example, EVs from cancer-associated fibroblasts carry transcription factors like SNAI1/ZEB1 or miRNAs (e.g. miR-193a, miR-210 from bone marrow cells) that prime lung cells for invasiveness (via STAT3, Wnt, TGF-β pathways) (Zhang et al., 2021; Fan et al., 2020).

Through these and other pathways (NF-κB, STAT3, PI3K/Akt, etc.), EV-delivered miRNAs orchestrate multiple aspects of lung cancer progression, including proliferation, invasion, immune suppression, and metastatic niche formation. Notably, many of these functions have been validated *in vitro* and in animal models, underscoring the causal role of EV-miRNA transfer in lung cancer pathogenesis and immune modulation (Iranpanah et al., 2023; Yoo et al., 2018).

Other examples of EV-miRNAs in lung cancer include miR-30e-3p, which is downregulated in chemoresistant NSCLC and functions as a predictive biomarker for cisplatin response through regulation of DNA damage repair pathways (Papadaki et al., 2025), and let-7e, a tumour-suppressive exosomal miRNA whose reduced expression correlates with metastasis and poor clinical outcome (Xu et al., 2021); Additional EV-miRNAs such as miR-451a and miR-486-5p serve as diagnostic and prognostic biomarkers distinguishing small-cell from non-small-cell lung cancer (Niu et al., 2025; Ding et al., 2021), while specific EV-miRNA panels have been described for adenocarcinoma and squamous carcinoma subtypes (Abdipourbozorgbaghi et al., 2024). In aggregate, dozens of tumour-derived miRNAs have been detected in blood EVs of lung cancer (Martínez-Espinosa et al., 2024). These miRNAs include oncogenic ones and tumour-suppressors that are dysregulated in cancer (Qian H. et al., 2024; Tulinsky et al., 2022; Ye et al., 2023). Specifically, exosomal miR-21 has been experimentally shown to promote macrophage M2 polarization and tumour progression in NSCLC by targeting IRF1 under hypoxic conditions (Jin and Yu, 2022). Tumour-associated EV-miRNAs in lung cancer are summarised in Table 2, organised by subtype and stage, clinical significance, and mechanistic pathways.

**TABLE 2:** Tumour-associated EV-miRNAs in lung cancer organised by subtype, stage, clinical significance, and mechanistic pathways.

EV-miRNA	Lung cancer type/stage	Clinical significance (diagnosis/prognosis/therapy)	Mechanistic pathway involved	Reference
miR-21	NSCLC, hypoxic and advanced stages	Diagnostic and prognostic biomarker; promotes invasion and tumour progression via immune modulation	Exosomal miR-21 targets IRF1, suppresses its expression, and induces macrophage M2 polarization under hypoxia	Jin and Yu (2022)
miR-155	NSCLC, metastatic	Promotes metastasis and EMT; associated with poor prognosis	M2 tumor-associated macrophage derived exosomal miR-155 directly targets RASSF4, suppressing its expression and enhancing tumour cell migration, invasion, and EMT	Li et al. (2021b)
miR-210-3p	NSCLC (circulating EVs, clinical samples)	Diagnostic and prognostic biomarker; elevated levels correlate with tumour progression and poor survival	Hypoxia-regulated miRNA induced by HIF-1α; associated with angiogenesis and metabolic reprogramming	Hassanin and Ramos (2024)
let-7e	NSCLC (Adenocarcinoma, early to advanced stages)	Tumour-suppressive EV-miRNA; its reduced expression in serum exosomes correlates with metastasis and poor prognosis; restoration inhibits migration and invasion	Regulates the SUV39H2/LSD1/CDH1 axis to suppress EMT and metastatic potential	Xu et al. (2021)
miR-30e-3p	NSCLC, chemoresistant/advanced stage	Downregulated in chemoresistant NSCLC; predictive biomarker for cisplatin response; involved in tumour suppression and DNA damage repair	Linked to p53, PI3K, and TGF-β signaling pathways	Papadaki et al. (2025)
miR-451a	NSCLC vs SCLC, metastatic	Downregulated in NSCLC; diagnostic and prognostic biomarker; correlates with metastasis and poor survival	Regulates MIF-mediated pathways controlling proliferation and migration	Niu et al. (2025)
miR-486-5p	SCLC vs NSCLC	Diagnostic biomarker distinguishing histological subtypes; associated with proliferation and apoptosis	Cell cycle regulation and apoptosis pathways	Ding et al., 2021
miR-30d-5p	Lung adenocarcinoma with lymphovascular invasion (early-stage)	Downregulated in exosomes of LVI-positive patients; prognostic biomarker indicating aggressive phenotype and poor recurrence-free survival	Regulates invasion, angiogenesis, and EMT-related pathways	Shimada et al. (2023)
miR-200 family	NSCLC, metastatic and early-stage	Downregulated in tumour tissues and plasma; correlated with metastasis and poor differentiation; strong diagnostic and prognostic biomarker	Regulates ZEB1/2 and E-cadherin pathways, inhibits EMT and metastasis	Ling and Yang (2024)
miR-126	NSCLC, advanced	Anti-angiogenic and prognostic biomarker; suppresses tumour growth	Inhibits PI3K/AKT pathway; suppresses VEGF expression	Wu et al. (2021)
miR-23a (miR-23a/27a/24-2 cluster)	NSCLC, early-stage (postoperative recurrence)	Promotes postoperative recurrence and metastasis; potential prognostic biomarker	Activates Wnt/β-catenin signalling; induces promoter methylation of p16 and CDH13	Fan et al. (2021)

Most evidence to date derives from *in vitro* and animal studies, underscoring the causal role of EV-miRNA transfer in lung cancer pathogenesis. Yet, a critical translational gap remains: whether circulating EV-miRNAs act as drivers of disease in humans or represent epiphenomena of tumour activity. Addressing this will require interventional studies targeting EV-miRNA pathways, which could clarify their dual value as biomarkers and therapeutic targets.

Given their dual role as both effectors of tumour progression and stable carriers of molecular cargo, EVs occupy a unique position where the same vesicles that drive oncogenic communication can also be harnessed as non-invasive reporters of tumour presence and behaviour.

### 3.4 Potential for early detection and non-invasive monitoring

Circulating EVs and their miRNAs show great promise as non-invasive lung cancer biomarkers. Because they circulate at high levels and protect their cargo, EV-based assays can sensitively detect tumour signals. For example, a recent study built a 5-miRNA EV signature from plasma that achieved AUC = 0.920 for distinguishing malignant from benign pulmonary nodules (training cohort) (Zheng et al., 2022). Other large studies report EV miRNA panels with sensitivities ∼75–85% and specificities ∼60–80% for early NSCLC detection. In one cohort of 330 NSCLC patients and 332 controls, combining serum exosomal miR-5684 and miR-125b-5p gave AUC 0.793 (sensitivity 82.7%, specificity 62.1%) (Zhang et al., 2020). Another study showed that adding EV miRNAs (miR-320a, miR-622) to serum CEA and CYFRA21-1 markers could distinguish metastatic vs. non-metastatic NSCLC with AUC ≈ 0.90 (Wang et al., 2020). These results demonstrate that EV-miRNA profiles can rival or complement existing tumour markers.

EV-based liquid biopsy is being evaluated in clinical settings. For example, a phase 2 trial (ChiCTR1800019877) used EVs from bronchoalveolar lavage fluid (BALF) of NSCLC patients: targeted EV-DNA/RNA analysis (ddPCR, NGS) identified EGFR driver and resistance mutations without tissue biopsy (Tulinsky et al., 2022; Rayamajhi et al., 2024). More broadly, high-throughput platforms (small-RNA NGS of EVs, digital PCR, and emerging microfluidic/nanotech devices) enable multiplexed EV biomarker measurement. Notably, advanced EV-isolation chips (e.g. AC-electrokinetic “Verita” systems) can rapidly enrich exosomes for downstream protein and RNA assays, pointing to scalable diagnostics (Hinestrosa et al., 2022).

Compared to traditional liquid biopsy analytes, EVs offer distinct advantages. Their lipid envelope renders cargo (miRNAs, mRNAs, DNA) more stable than unprotected cell-free nucleic acids. EV assays can capture a broader spectrum of tumour material (RNA and protein in the same vesicle) than ctDNA alone. Moreover, because EVs are continuously shed by tumours, they may detect cancer signals even when ctDNA levels are low (e.g. early-stage disease). However, challenges remain such as EV isolation lacks standardisation and can co-isolate non-tumour vesicles or lipoproteins, potentially reducing specificity. Analytical variability (yield/purity) and the heterogeneous origins of EVs in blood can complicate interpretation (Irmer et al., 2023; Phillips et al., 2021). Nonetheless, EV-based tests enable truly repeatable “liquid biopsies” with minimal patient risk.

In summary, circulating EVs are emerging as a powerful liquid-biopsy source for lung cancer. They can harbour sensitive signatures of tumour miRNAs and proteins, with multiple studies reporting promising diagnostic metrics (high AUC, sensitivity/specificity). Ongoing trials and improved technologies (NGS, ddPCR, nano-capture chips) are advancing EV assays toward clinical use. Compared to tissue biopsy or cfDNA tests, EV-based biomarkers offer non-invasive, multi-dimensional insights into tumour genetics and biology, although further validation and standardisation are needed before routine deployment.

## 4 Technological approaches for EV isolation and miRNA profiling

### 4.1 Isolation techniques for EVs: principles, advantages, and limitations

Considering their diverse functions and promising clinical applications, achieving high yields and quality of sEVs is crucial. Numerous isolation methods have been developed, primarily based on the biophysical and biochemical properties of EVs, such as size, density, shape, and surface markers. The choice of isolation technique should be guided by both the intended application whether diagnostic or therapeutic, and the complexity of the biological fluid from which the EVs are derived (Jia et al., 2022).

EVs have attracted significant attention over the past few decades as potential drug delivery vehicles and biomarkers for a wide range of diseases. However, a major challenge in advancing their broader application lies in selecting an optimal, efficient, and reliable isolation strategy. Commonly used techniques include filtration, ultracentrifugation, and affinity-based separation. To obtain well-purified and functionally intact vesicles, it is often necessary to use a carefully selected combination of isolation and purification methods (Akbar et al., 2022). Beyond these approaches, additional strategies have been increasingly employed in recent years. Density gradient centrifugation provides higher purity by separating EVs from lipoproteins and protein aggregates, though it is labour-intensive (Greening et al., 2015). Size-exclusion chromatography (SEC) is considered gentle and reproducible, preserving vesicle integrity but producing diluted fractions (Monguió-Tortajada et al., 2019). Precipitation-based methods, such as PEG-mediated precipitation, are simple and scalable but often co-isolate protein contaminants (Wang P. et al., 2023). Immunoaffinity capture allows enrichment of EV subpopulations using antibodies against markers like CD9, CD63, and CD81, yet yields are typically low and costs high (Fan et al., 2023). More recently, microfluidic-based devices that integrate acoustic, immunoaffinity, and electrophoretic principles have enabled rapid and high-throughput EV isolation from small sample volumes, holding strong potential for clinical translation, though large-scale validation is still required (Hassanzadeh-Barforoushi et al., 2025; Dai et al., 2025).

Numerous techniques have been developed for the isolation of EVs; however, none have yet achieved complete removal of contaminants that may compromise downstream analyses. While innovative and improved methodologies continue to emerge, their broader adoption remains limited due to the need for specialised expertise and the high associated costs (Stam et al., 2021). Table 3 provides a comprehensive overview of both conventional and recently developed methods for the isolation of EVs. Each technique is described by its underlying principle, along with key advantages and limitations relevant to research and clinical applications.

**TABLE 3:** Comparison of EV isolation methods: Principles, advantages, and limitations.

Method	Principle	Advantage	Disadvantage	References
Differential Ultracentrifugation	Sequential centrifugation at increasing speeds to pellet EVs by size and density	Widely used; enables processing of large volumes	Time consuming; low purity; potential EV damage	Théry et al. (2006), Livshits et al. (2015)
Density Gradient Ultracentrifugation	Separation based on buoyant density using a medium (e.g., sucrose or iodixanol)	Higher purity than differential ultracentrifugation	Larger volume required, low yield, time-consuming	Kowal et al. (2016), Akbar et al. (2022)
Size-exclusion Chromatography (SEC)	Separation based on size using porous beads	Preserves EV integrity; fast and simple	Limited sample volume per run; co-isolation of similarly sized particles	Böing et al. (2014), Baranyai et al. (2015)
Ultrafiltration	Based on size exclusion using membrane filters with defined molecular weight cut-offs (typically 100–500 kDa or 10–200 nm pore sizes)	Easy to perform	Considerably influences EV yield and composition	Heinemann et al. (2014), Stam et al. (2021)
Polymer-Based Precipitation (e.g., PEG)	EVs precipitated using polymers (e.g., polyethylene glycol)	Easy, cheap and fast; high EV yield	Low purity; co-precipitation of proteins and other contaminants	Rider et al. (2016), Stam et al. (2021)
Immunoaffinity Capture	Uses antibodies against EV-specific surface markers (e.g., CD63, CD81)	High specificity and purity	Expensive; limited yield	Tauro et al. (2012), Stam et al. (2021)
Microfluidic Devices	Isolation via miniaturised platforms integrating size, affinity, or acoustic principles	Can be combined with EV characterisation; Rapid processing; requires small sample volume	Requires specific expertise	Liga et al. (2015), Chen et al. (2019), Stam et al. (2021)
Field-Flow Fractionation (FFF)	Separation based on differences in particle mobility in a flow field perpendicular to the main channel flow	Capable of identifying smaller EV (exomeres)	Can be used as a stand-alone method, complex and needs expertise	Zhang et al. (2018), Contado (2017)
Membrane Affinity	Uses membranes or surfaces functionalised to selectively bind EVs based on affinity interactions	Isolate all EVs	Cannot distinguish between EVs and cellular fragments	Ghosh et al. (2014), Konoshenko et al. (2018), Stam et al. (2021)
FLOAT (FLocculation via Orbital Acoustic Trapping)	Uses acoustofluidic droplet centrifugation and thermo-responsive flocculants to trap and concentrate EVs	High yield (>90%), minimal sample required (∼200 µL), protein contaminant removal	Requires acoustofluidic expertise and setup	Rufo et al. (2024)
Fe_3_O_4_@ZrO_2_ Magnetic Bead-Based Isolation	Magnetic beads coated with ZrO_2_ selectively bind EVs via phosphate interactions	High purity (∼97%), fast (30 min), and reproducible (0.3%–0.5%)	Dependence on bead quality and cost, sensitive to operating conditions; challenging sample recovery	Yang et al. (2024)
EVics (Extracellular Vesicle Isolation and Counting System)	An integrated system designed for high-efficiency EV isolation and quantification across volume ranges	Isolates and quantifies EVs from 15 µL to 2 L, high purity and faster	Specialised Equipment Requirements	Bae et al. (2024)

### 4.2 Analytical methods for profiling miRNAs in EV

The identification of miRNAs within EVs is critical for elucidating mechanisms of intercellular communication and advancing their application as diagnostic biomarkers (Xu D. et al., 2022). A range of methodologies has been developed for the qualitative and quantitative profiling of miRNAs in EVs, each characterised by distinct principles, strengths, and limitations. Among these, quantitative reverse transcription polymerase chain reaction (RT-qPCR) remains the most widely employed technique due to its high sensitivity and specificity for known miRNA targets. In addition, several other analytical strategies have been established, including molecular beacon *in situ* hybridisation (MB *in situ*), surface-enhanced Raman scattering (SERS), microarray analysis, next-generation sequencing (NGS), and isothermal amplification method (Xu M. et al., 2022) (Table 4).

**TABLE 4:** Summary of advantages and disadvantages of miRNA detection methods.

Method	Advantages	Disadvantages	References
RT-qPCR	High sensitivity	Time consuming	Jiang et al. (2021b)
MB *in situ*	Simple method	False-negative caused by low concentration	Lee et al. (2018)
SERS	High sensitivity, fast, and simple	Special equipment	Pang et al. (2019)
Microarray	Detect many sequences	Low sensitivity, cost and	Nakamaru et al. (2020), Tan et al. (2021)
NGS	High sensitivity and accuracy	Cost and complex data analysis	Selmaj et al. (2017)
Isothermal amplification method	No heating for amplification	Complex primer design	Deng et al. (2017)

#### 4.2.1 Quantitative reverse transcription polymerase chain reaction (RT-qPCR)

The gold standard method that is most commonly used for detecting miRNA in EVs. It depends on two stages: first is the synthesis of complementary DNA by reverse transcriptase (RT), followed by using cDNA for PCR amplification. The amplification process is monitored in real time by measuring fluorescence signals emitted either from a double-stranded DNA-binding dye or a sequence-specific fluorescent probe (Xu Y. et al., 2022). In lung cancer research, RT-qPCR has been widely applied to quantify circulating EV-miRNAs such as miR-21, miR-155, and miR-210-3p, which are associated with tumour progression, metastasis, and poor prognosis (Jin and Yu, 2022; Li X. et al., 2021; Hassanin and Ramos, 2024). To solve the challenge of low sensitivity, droplet digital PCR (ddPCR) was designed. ddPCR showed higher accuracy and reproducibility for serum miRNA and can detect low copy numbers of nucleic acids, however, it requires special equipment and is costly (Hindson et al., 2013). In addition, high-throughput RT-qPCR platforms such as Fluidigm BioMark, together with the use of locked nucleic acid (LNA)-modified primers, have enhanced sensitivity and multiplexing capacity, enabling robust detection of low-abundance EV-miRNAs from minimal sample volumes (Takousis, 2018; Jang et al., 2011; He et al., 2021).

#### 4.2.2 Molecular beacon *in situ* (MB *in situ*)

The molecular beacon (MB) is a nanoscale, bi-labelled oligonucleotide probe structured as a hairpin loop, featuring a fluorescent reporter dye at one end and a quencher molecule at the opposite end. The resulting fluorescence intensity is directly proportional to the concentration of the MB–miRNA hybrid (Lee et al., 2015). It is a simple process that does not need RNA extraction or amplification, it depends on sample incubation with the beacon. The drawback of this method is the false negative that is caused by low concentration (Lee et al., 2018). To address these limitations, nanomaterial-assisted probes such as graphene oxide and quantum dots have been developed to enhance signal-to-noise ratios and reduce false negatives. Furthermore, microfluidic integration of MB assays has enabled automated, high-throughput detection of EV-miRNAs in clinical samples (Oliveira et al., 2020; Lee et al., 2016). In the context of lung cancer, MB-based approaches have been applied for detecting EV-associated miRNA signatures in patient-derived samples, offering rapid and sensitive monitoring of disease-related molecular changes. This highlights their potential as non-invasive diagnostic and prognostic tools specifically tailored to lung cancer.

#### 4.2.3 Surface-enhanced Raman scattering (SERS)

An increasing number of studies are focusing on the detection of EV-derived miRNAs (miRNAs) using surface-enhanced Raman scattering (SERS) techniques. A recent study demonstrated the use of a modified dual SERS biosensor for the sensitive detection of miRNAs isolated from exosomes. The biosensor incorporates a SERS label consisting of Fe_3_O_4_@Ag-DNA-Au@Ag@DTNB nanoparticles, functionalised with DNA oligonucleotides complementary to the target miRNA. Upon hybridisation, the target miRNA forms a duplex with the complementary DNA, which is subsequently recognised and cleaved by a duplex-specific nuclease. This cleavage event leads to the release of the SERS-labelled nanoparticles from the substrate, resulting in a measurable quenching of the Raman signal (Pang et al., 2019). More recent SERS platforms allow multiplexed detection of multiple EV-miRNAs simultaneously, and the incorporation of machine learning-assisted spectral analysis has enhanced diagnostic accuracy and sensitivity, supporting their application in clinical liquid biopsy (Neettiyath et al., 2024; Xu et al., 2025).

#### 4.2.4 Microarray

Microarray analysis relies on the use of pre-designed labelled probes that hybridise specifically with complementary cDNA sequences. In this approach, total RNA is first extracted from EVs isolated from the biological sample. Subsequently, a complementary DNA (cDNA) library is synthesised from the extracted RNA. For miRNA detection, the resulting cDNA is incubated with immobilised probes on the microarray chip. Hybridisation occurs between the target cDNA and the corresponding labelled probes, and the relative expression levels of miRNAs are quantified based on the intensity of the resulting hybridisation signals (Sevenler et al., 2018; Li P. et al., 2020). In lung cancer studies, EV-derived miRNA microarray profiling has enabled the identification of diagnostic signatures capable of distinguishing malignant from benign pulmonary nodules, supporting its value in liquid biopsy approaches.

#### 4.2.5 Next-generation sequencing (NGS)

NGS is a high-throughput technology widely used in transcriptome analysis (Miller et al., 2022). It consists of several steps starting from RNA is first extracted and purified, followed by the ligation of universal adaptors to the 5′ and 3′ ends. Reverse transcription and PCR amplification are then performed before sequencing. Compared to microarrays, NGS has superior sensitivity and can overcome the limitations of microarrays, such as the need for large sample quantities and the inability to detect unknown miRNA (Xu D. et al., 2022). When applied to lung cancer, NGS-based EV-miRNA profiling has uncovered novel miRNA biomarkers linked to tumour stage and prognosis, offering higher resolution for early detection and disease monitoring.

#### 4.2.6 Isothermal amplification methods

Isothermal amplification has emerged as a promising alternative to polymerase chain reaction (PCR) for the rapid and efficient amplification of nucleic acids. Unlike PCR, isothermal amplification operates at a constant temperature, enabling nucleic acid amplification under simplified conditions, such as in a water bath. Since its introduction in the early 1990s, a variety of isothermal amplification methods have been developed, offering rapid, sensitive, and straightforward approaches for nucleic acid detection without the need for specialised thermal cycling equipment (Zhao et al., 2015). Isothermal amplification is divided into enzymatic isothermal amplification, that based on exponential or linear amplification kinetics and includes loop-mediated isothermal amplification (LAMP), nuclear acid sequence-based amplification (NASBA), rolling circle amplification (RCA), exponential amplification reaction (EXPAR), and duplex-specific nuclease amplification reaction (DSNAR) and enzyme-free isothermal amplification that relies on competitive hybridisation, including catalytic hairpin assembly (CHA) and hybrid chain reaction (HCR) (Gines et al., 2020).

### 4.3 Challenges in miRNA profiling from plasma- and serum-derived EVs

The most common human biofluids that have been commonly used for EVs isolation are plasma and serum. The major significant challenge that is associated with these samples is the availability of limited volume, which results in low yield of RNA that could affect the downstream applications, for example, that 2 mL aliquot of serum yields about 200 pg/µL of RNA leading to a reduction in miRNA profiling analyses (Chen et al., 2022). Additionally, the type of isolation method affects the yield of miRNA (Gámez-Valero et al., 2016). Furthermore, heterogeneity of RNA in EVs is considered a problem, as some researchers have suggested that the sEVs lack miRNA enrichment (Chen et al., 2022; Li C. et al., 2021), while others suggest that sEVs carry more tRNA (Chevillet et al., 2014; Hoen et al., 2012). Also, current RNA quality control standards were originally developed for cellular RNA analyses; this method does not represent the RNA cargo that is typically found in sEVs (Valadi et al., 2007; Bellingham et al., 2012; Murillo et al., 2019). Another limitation is the technical variability for effective RNA quantification that requires establishing references and processing controls before downstream computational processing (Tesovnik et al., 2021). Furthermore, the RNA subtypes in EVs are different from cellular small RNA (originally optimised for cellular RNA) may be suboptimal for EV-derived RNA (Chen et al., 2022; Eldh et al., 2012). Moreover, the accuracy of RNA mapping, size distribution profiles and transcript abundance estimation are affected by the selection of sequencing aligners and annotation databases; as a result, analysing EV transcriptomics is more challenging than traditional cellular transcriptomics (Padilla et al., 2023; Everaert et al., 2019).

Collectively, these challenges highlight the urgent need for standardised protocols, EV-specific quality control measures, and tailored analytical approaches to ensure the accuracy, reproducibility, and clinical relevance of EV-derived miRNA profiling.

### 4.4 Artificial intelligence and next-generation analytical tools

Integration of artificial intelligence (AI) with microfluidic platforms has been shown to enhance EV isolation and analysis. A deep learning–based on-chip system demonstrated automated image identification of tumour exosomes, reducing manual intervention and increasing throughput. Such approaches improve sensitivity, reproducibility, and workflow efficiency, though challenges remain regarding dataset quality, generalisability, and model interpretability (Lu et al., 2025). Beyond isolation, machine learning has also been applied to EV analysis in clinical contexts such as transplant monitoring, where EV-derived molecular features combined with AI enabled earlier and more accurate detection of graft rejection than conventional biomarkers. Although broader validation is needed, this illustrates the potential of AI to extend the diagnostic utility of EVs (Burrello et al., 2025). In oncology, machine learning similarly enhances EV analysis by integrating complex molecular data to improve classification, early detection, and treatment monitoring. Despite these advances, progress is limited by variability in isolation methods, small cohort sizes, and interpretability challenges, underscoring the need for standardised approaches and large-scale validation (Greenberg et al., 2023). Looking ahead, AI also shows promise in advancing EV-based precision drug delivery by optimising isolation, loading, and therapeutic targeting. However, translation into clinical practice will require overcoming constraints related to dataset quality, model transparency, and regulatory approval (Greenberg et al., 2023).

Recent advances have sought to overcome the challenges of EV heterogeneity and limited sensitivity in miRNA-based diagnostics. Deep learning has recently been applied to enhance miRNA profiling within single EVs for cancer diagnosis. By combining fluorescence imaging with AI-based classification, this approach enables multiplexed detection of miRNA signatures at the single-vesicle level, improving sensitivity and revealing EV heterogeneity. Such methods hold promise for more accurate liquid biopsy applications, though further validation and standardisation are required for clinical translation (Zhang et al., 2024). Similarly, a dual-surface-protein orthogonal barcoding strategy has been developed to enable simultaneous tracing of exosome subsets and profiling of their miRNA cargo. By linking surface protein identity with molecular signatures, this approach provides greater resolution of exosome heterogeneity and improves specificity in distinguishing tumour-derived exosomes. Such methods offer valuable insights for cancer diagnostics, though their complexity and need for clinical validation remain important limitations (Lei et al., 2023).

## 5 EV-mediated miRNA therapeutics: strategies, applications, and clinical translation

EVs are being engineered as biocompatible carriers for miRNA mimics and antimiRs to modulate oncogenic pathways in lung cancer. Preclinical studies demonstrate that EVs can protect miRNA cargo from nuclease degradation, improve cellular uptake, and enable functional delivery to tumour and stromal cells, offering advantages over synthetic nanoparticles. Recent reviews outline multiple lung-relevant targets with roles in proliferation, EMT, angiogenesis, and immune evasion, positioning EVs as promising vectors for these payloads (Munir et al., 2020; Poongodi et al., 2025).

Loading approaches include producer-cell overexpression, electroporation, sonication, saponin permeabilisation, and chemical transfection; each has trade-offs in loading efficiency and vesicle integrity. For tissue targeting, surface display and antibody/aptamer decoration are under active development to increase tumour tropism and reduce off-target exposure (Seyhan, 2023).

Growing experimental evidence supports EV-mediated restoration of tumour-suppressive miRNAs or inhibition of oncogenic miRNAs in thoracic oncology models. For instance, EV-delivered miR-200 family members (miR-200a/b/c, miR-141, and miR-429) suppress EMT and metastatic behaviour in NSCLC by targeting ZEB1/2 and restoring E-cadherin, as confirmed in recent experimental studies (Ling and Yang, 2024). Likewise, EV-miR-126 has demonstrated anti-angiogenic and anti-metastatic effects, while cancer-associated fibroblast–derived EVs were shown to regulate metastatic phenotypes through miR-200 signalling axes. Although most findings remain preclinical, these studies collectively substantiate EVs as modular carriers capable of reprogramming oncogenic circuits in NSCLC (Romanò et al., 2024).

EV cargo can modulate response to immune checkpoint blockade. Beyond PD-L1 transfer, miRNA payloads that down-tune immunosuppressive pathways may synergise with anti-PD-1/PD-L1 by reshaping T-cell states and the cytokine milieu. Lessons from the first-in-human miRNA mimic trial underscore the need for careful dosing, immune monitoring, and delivery-platform selection after immune-related toxicities led to early termination; EVs are being explored as potentially less immunogenic carriers to revisit such tumour-suppressor miRNAs (Brillante et al., 2024).

Clinical deployment requires standardised manufacturing, release testing, and potency assays. Recent consensus and regulatory-facing guidance emphasise identity, purity, reproducibility, and mechanism-of-action–linked potency metrics across the product life cycle. Parallel efforts track early clinical experience with EV-based interventions and map translational hurdles, scalable production, batch-to-batch consistency, and validated bioassays to accelerate oncology applications (Morse et al., 2005; Ng et al., 2022).

EV-based miRNA therapeutics show strong potential to modulate oncogenic pathways in lung cancer, offering stability and specificity over conventional systems. While preclinical results are promising, clinical translation requires overcoming challenges in safety, manufacturing, and standardisation.

## 6 Conclusion and future prospects

EVs and their cargo of miRNA molecules represent a rapidly advancing frontier in the non-invasive diagnosis and management of lung cancer. The exceptional stability of EV-carried miRNAs in the circulatory system, along with their cancer-specific expression signatures, make them highly attractive candidates as liquid biopsy biomarkers. Their detection from easily obtainable biological fluids such as blood, saliva, or bronchoalveolar lavage offers practical advantages in terms of patient comfort, cost-effectiveness, and clinical applicability. EV-associated miRNAs have demonstrated significant potential not only in the early detection of NSCLC, but also in the prediction of disease progression, monitoring of therapeutic response, and even in stratifying patients based on likely prognosis. Importantly, these miRNAs may provide real-time insights into tumour evolution and resistance mechanisms, enabling clinicians to dynamically adjust treatment regimens. Despite the promising outlook, several challenges remain. There is a critical need for standardization of EV isolation and miRNA profiling protocols, as well as robust validation across large, multi-centre cohorts to ensure reproducibility and clinical reliability. Furthermore, deciphering the molecular mechanisms through which EV-derived miRNAs regulate tumour initiation, angiogenesis, metastasis, and immune evasion will be essential for their integration into precision oncology. Looking ahead, EVs also offer exciting possibilities as therapeutic vectors, capable of delivering miRNAs or gene-silencing agents directly to tumour cells with high specificity. This opens the door to a new generation of miRNA-based therapeutics, potentially overcoming limitations of conventional treatments by targeting cancer at the epigenetic and post-transcriptional level. Unlike previous reviews that primarily emphasized the diagnostic value of EV-miRNAs, this review provides a broader and updated synthesis by integrating recent insights into EV–miRNA–mediated signaling, unresolved challenges such as heterogeneity and standardisation, and forward-looking perspectives including single-EV detection, multi-omics integration, AI-driven analytics, and therapeutic applications. In conclusion, EV-associated miRNAs stand at the crossroads of diagnostics and therapeutics, offering a unique dual role as both biomarkers and biological modulators. Continued interdisciplinary research combining molecular biology, bioinformatics, and clinical oncology will be essential to translate these insights into tangible benefits for patients with lung cancer. Future prospects include the integration of EV-miRNA profiling with multi-omics approaches (genomics, proteomics, metabolomics) to create more comprehensive biomarker panels for precision medicine. Advances in microfluidics and single-vesicle sequencing may allow high-resolution analysis of miRNA heterogeneity, thereby improving diagnostic specificity. Moreover, the convergence of AI with EV research holds promise for automating biomarker discovery, predicting therapeutic responses, and personalising treatment regimens. Clinically, EV-based delivery systems for engineered miRNAs or gene-silencing tools may emerge as a new generation of targeted therapeutics, though their safety, scalability, and regulatory approval remain key challenges. Ultimately, collaborative efforts across basic science, clinical research, and bioengineering will be required to move EV-miRNAs from bench to bedside and establish them as mainstream tools in lung cancer management.

## Abbreviations

ApoBD, Apoptotic Body; ddPCR, Droplet Digital PCR; ESCRT, Endosomal Sorting Complex Required for Transport; EVs, Extracellular Vesicles; LEV, Large Extracellular Vesicles; miRNAs, microRNAs; MVBs, Multivesicular Bodies; NGS, Next-Generation Sequencing; PS, Phosphatidylserine; RT-qPCR, Reverse Transcription quantitative Polymerase Chain Reaction; SERS, Surface-Enhanced Raman Scattering; SEV, Small Extracellular Vesicles.
